# The effect of sage (Salvizan gel) compared to triamcinolone acetonide on the treatment of recurrent aphthous stomatitis: a double-blinded randomized clinical trial

**DOI:** 10.1186/s12903-023-02861-y

**Published:** 2023-03-18

**Authors:** Fatemeh Abbasi, Zeinab Rasoulzadeh, Amirmohammad Yavari

**Affiliations:** 1 Department of Oral and Maxillofacial Medicine, Dental Research Center, Dental Research Institute, School of Dentistry, Isfahan University of Medical Sciences, Isfahan, Iran; 2 Student Research Committee, School of Dentistry, Isfahan University of Medical Sciences, Isfahan, Iran

**Keywords:** Recurrent aphthous stomatitis, Salvizan gel, Triamcinolone acetonide gel

## Abstract

**Background:**

Recurrent Aphthous Stomatitis (RAS) is one of the most common lesions of the oral mucosa. Herbal medicine can be used for the treatment of this disease. The present study aimed to compare the effects of topical application of Sage (Salvizan) and triamcinolone acetonide gels on RAS.

**Methods:**

This double-blind clinical study recruited sixty patients with minor aphthous ulcers. Half of the patients were treated with Salvizan gel, and the other half were treated with oral triamcinolone acetonide gel. The effect of Salvizan topical gel was evaluated and compared with that of oral triamcinolone acetonide gel. Factors such as Pain recovery time, wound healing time, and pain level was evaluated. Data were analyzed by SPSS version 22 using independent t-test, paired t-test, repeated measures ANOVA, and survival analysis, including Kaplan-Meier and Cox regression.

**Results:**

The mean duration of pain recovery was 1.5 days for Salvizan and 2.5 days for triamcinolone acetonide (*p* < 0.001). Moreover, the duration of wound healing was 3.3 days for Salvizan and 6 days for triamcinolone acetonide (*p* < 0.001). Patients’ satisfaction from factors such as taste and smell had no significant difference between the two groups.

**Conclusion:**

The results of this study showed that Salvizan gel is very effective in the treatment of RAS. It was significantly better than triamcinolone acetonide in the pain recovery and wound healing. These promising results favor herbal treatments and show that they can be used more commonly for treating diseases such as RAS.

**Trial registration:**

This study was approved by the ethics committee of Isfahan University of Medical Sciences (IR.MUI.RESEARCH.REC.1399.834) on 17/03/2021. It was also registered in the Iranian Registry of Clinical Trials (IRCT20100202003251N7).

## Background

Recurrent Aphthous Stomatitis (RAS) is a common oral mucosa disease characterized by single or multiple painful and recurrent ulcers confined to the oral cavity. Its prevalence in different populations has been reported to vary from 5 to 50%, with an average prevalence rate of 20% and higher occurrence in young women [1]. The prevalence of RAS in the Iranian population was estimated to be 20–26%, which is a high rate [2, 3]. Aphthous ulcers are hurtful for patients and cause pain and burning when eating, swallowing, and talking. These problems interrupt the functions of the patients and significantly reduce the quality of life in both children and adults [4, 5].

There are predisposing factors reported for this disease, such as psychological, hematological, genetic, traumatic, and allergic factors. Still, the etiology of the disease is unknown. So, there is no definitive cure for the disease [6, 7]. While the RAS is active, the treatment’s main goal is to minimize the symptoms and delay the recurrence after the healing. Treatments of RAS are supposed to relieve pain and burning, reduce the disease period, and delay the recurrence of ulcers [7, 8]. Various pharmaceutical compounds in the form of topical and systemic have been developed for the treatment of RAS. Among topical drugs, various options are available, including antiseptics, anti-inflammatories, antibiotics, and corticosteroids [6]. A successful and commonly used group for this purpose is the topical corticosteroids. Triamcinolone acetonide is a moderate steroidal drug used to treat inflammatory and allergic disorders, including RAS [9]. Although short-term use of corticosteroids usually does not cause side effects, their long-term use can cause local and systemic side effects such as candida superimposition, adrenal suppression, mucosal thinning, drug resistance, Cushing’s syndrome, and osteoporosis [10].

Herbal medicines have become prevalent among people because of several health benefits attributed to these natural and safe agents. Herbal medicines are also widely used in dentistry because of their unique properties, including antimicrobial, antifungal, anti-inflammatory, and antioxidant effects [11]. Due to the popularity of herbal medicine and its antimicrobial and anti-inflammatory effects, much research has been conducted on the effect of herbal medications on treating different diseases. One example is RAS, in which various herbal products have been introduced for its treatment. Studies have investigated various types of herbal treatments, such as curcumin [12], coconut [13], aloe vera [14], myrtle [15], licorice [16], Sicilian sumac [17], and pomegranate [18] on treating aphthous ulcers.

Salvia officinalis (common Sage, culinary Sage) is an aromatic plant often used for different purposes, especially as a traditional medicine for treating several infectious diseases [19]. Sage contains different compounds with influential characteristics. Ethanolic compounds of Sage have antibacterial activity [20]. Also, the terpenoids available in leaves of Sage, such as ursolic acid, have an anti-inflammatory effect [21]. This effect has led to the design and manufacture of potent drug molecules from this plant extract to treat chronic inflammatory diseases [22]. Additionally, its flavonoids and phenolic acids have antioxidant properties and protect against viral and bacterial infections in vitro and in vivo [23].

Salvizan gel is a pharmaceutical product made of Sage extract used to alleviate the inflammation of the oral mucosa. A study in 2021 compared the effects of Salvizan and triamcinolone acetonide gels on the duration and symptoms of oral lichenoid reactions. They reported that Salvizan gel significantly relieved the pain better than triamcinolone acetonide, which is promising for using Salvizan gel in treating oral lesions [24]. Also, in another study, the effect of Salvizan Gel was compared with Triadent (triamcinolone acetonide) in patients with minor aphthous ulcers in an Iranian population in the south of Iran. They concluded that both drugs are efficient for aphthous ulcers; Salvizan had a better performance for pain control, and Triadent had a better performance for reduction of the size of the ulcers [25].

Due to the antimicrobial, anti-inflammatory, antifungal, and analgesic effects of Sage, which is used in Salvizan gel, and the lack of evidence in the considered population, the present study aimed to evaluate the effect of Salvizan gel on minor oral aphthous ulcers in comparison to triamcinolone acetonide.

## Methods

### Study design and ethical approvement

This study was approved by the ethics committee of Isfahan University of Medical Sciences (IR.MUI.RESEARCH.REC.1399.834) on March 17th, 2021. All methods were performed in accordance with the relevant guidelines and regulations. It was a double-blind, randomized clinical trial conducted from May to June 2021. The study was also registered in the Iranian Registry of Clinical Trials (IRCT20100202003251N7) on 13/10/2021. Informed consent was obtained from patients involved in the study. The ethical guideline for this study was “General Ethical Guidance for Medical Research with Human Participants in the Islamic Republic of Iran.”

### Patient selection

This study was performed in the Dentistry school of the Medical University of Isfahan. Patients with confirmed RAS aged 18–50 were included in this study. Exclusion criteria were pregnant or lactating women, history of allergy to herbal products, history of a systemic disease, history of using anti-inflammatory or corticosteroids, presence of herpetiform and major aphthous ulcers, and presence of lesions for more than 48 h. With a standard deviation of 1.67, absolute precision of 1.2, an α of 0.95, and a β of 0.2, the sample size was calculated as 30 persons in each group. Accordingly, 60 eligible patients were recruited for the study, and after giving the necessary explanations about the study, informed consent was obtained from all patients before inclusion in the study. For the blindness of the study, a code (“A” or “B”) was assigned to each patient randomly to determine the patient’s drug. One of the codes belonged to triamcinolone acetonide (Triadent®, Raha Pharmaceutical Company, Isfahan, Iran), and the other belonged to Salvizan gel (Goldaru Herbal Pharmaceutical Company, Isfahan, Iran). Both are drugs introduced for the treatment of aphthous ulcers. Each patient was assigned to one group with a random process performed by a third person. This study had a parallel arm design. The drugs were randomly defined as “A” and “B”. Then 60 drug tubes were coded and divided into groups of A (n = 30) and B (n = 30) with an allocation ratio of 1:1. The relevant tube was given to each patient, with a covered tube, so the researchers were blind to the treatment of each group until the end of the study. The person who analyzed the results was also blind to the groups. At the end of the study, experiment and control groups were indicated using these codes.

### Treatment process

Patients were instructed on how to take the drug. Then they were asked to show the process to be sure they understood the instructions correctly. It was topically applied to aphthous ulcers three times a day using a swab as much as 1 cm, preferably after eating and mouth rinsing. After applying the drug, the patient was told not to consume any drinks or food for at least one hour and not to smoke. The patient consumed the drug until the pain was relieved and the ulcer healed completely. Anti-inflammatory and analgesic drugs should not be used simultaneously with this drug, either topically or systemically. Each patient was also given a card to record the pain score of the aphthous lesion. Patients were told to rate the pain and burning three times a day (in the morning, in the afternoon, and at night) for a week. For this purpose, Visual Analogue Scale (VAS) was used to determine the extent of the pain. With this method, the patient will rate the pain in a range of 0 to 10, depending on the amount of pain. On this scale, 0 indicates no pain, and 10 indicates maximum pain. Patients record their scores by marking the score on the graded line of the card. There were 21 lines with a specific date and time (i.e., Thursday, May 27th, morning).

Further, they had to record the day when the pain was completely relieved. Patients were recalled for several sessions on days 2, 4, 6, and 8 after receiving the treatment, to reexamine the lesion and ensure compliance with the instructions and the healing process of the lesion. When the pain and burning disappeared, the false membrane was removed. There should have been no ulcer, and the lesion was healed completely, which was recorded on the patient’s card.

### Statistical analysis

All data were fed into SPSS version 26 (software from IBM corp.) and analyzed by independent t-test, paired t-test, ANOVA for repeated measures, and survival analysis, including Kaplan-Meier and Cox regression. A significant level of *p* ≤ 0.05 was considered for the statistical analyses.

## Results

Among those referred to the designated center, 75 eligible patients were recruited for the study with full consent. Fifteen patients left the study for reasons such as being far away from the dentistry school (3 patients) and unwillingness to continue the treatment (12 patients). So, the study was performed with 60 patients, of whom 36 were women (60%), and 24 were men (40%), with an age range of 18–56 years. The chi-square test showed no significant difference in the frequency of gender between the two groups (*p* = 0.297). Both experimental groups included 30 patients (50%).

Data on the duration of pain and complete recovery are described in Table 1. Due to the lack of normality in both groups, the non-parametric Mann-Whitney test was used to compare the duration of pain recovery and complete recovery between the two groups. The results showed a significant difference between the two groups in pain and complete recovery (*p* < 0.001). For a more detailed analysis, survival analysis (Kaplan-Meier) was performed, which showed a significant difference between the two groups in terms of the duration of pain recovery and also complete recovery (*p* < 0.001).

**Table 1:** Mean duration of pain recovery and complete recovery of two drugs by days

Drug		Pain recovery	Complete recovery
Triamcinolone acetonide	Mean	2.5	6
	Number	30	30
	SD	0.81	1.01
	Minimum	1	4
	Maximum	5	8
Salvizan	Mean	1.5	3.3
	Number	30	30
	SD	0.9	1.14
	Minimum	1	2
	Maximum	4	8
Total	Mean	2	4.6
	Number	60	60
	SD	1	1.74
	Minimum	1	2
	Maximum	5	8

Most patients (about 63%) were satisfied with the smell of both drugs, about 20% had no opinion, and about 7% were dissatisfied. Among the patients, about 88.33% reported that they were satisfied with the form of both drugs, 8.33% had no opinion, and 3.33% were dissatisfied. The results showed that about 60% of patients were satisfied with the taste of both drugs, 20% were dissatisfied, and 20% expressed no opinion. Information obtained showed that all patients were satisfied with the treatment outcome. The Mann-Whitney test was used to evaluate satisfaction with factors such as smell, taste, form of consumption, and treatment outcome in both groups. Results showed no significant difference between them in taste (*p* = 0.825), smell (*p* = 0.272), satisfaction with the form of consumption (*p* = 0.350), and satisfaction with the treatment outcome (*p* = 0.394).

## Discussion

The present study showed promising results in treating RAS with Salvizan gel containing an extract of Sage. Compared to Triamcinolone acetonide, Salvizan gel performed better in both reducing the pain recovery and wound healing (complete recovery) times. Recurrent aphthous stomatitis is one of the most common oral diseases in the world and Iran, and still there is no definitive cure due to the unknown etiology. Results of this study show that Salvizan gel can be considered as a useful treatment of aphthous ulcers.

Considering the problems with chemical drugs, and increasing popularity of herbal treatments, many studies have been conducted to investigate their therapeutic effects on diseases such as RAS. Mainly they obtained promising results in favor of different herbal medicines such as curcumin [12], coconut [13], aloe vera [14], myrtle [15], Sicilian sumac [17], and Punica granatum (pomegranate) [18]. In many of these studies, the control group received triamcinolone acetonide, a corticosteroid that is a common drug for the treatment of aphthous ulcers. In some studies [12, 13, 15, 17] the herbal treatment and triamcinolone acetonide were both effective for the treatment of RAS, without any significant difference. But in other studies [14, 18] same as this study, the herbal treatment was significantly better than triamcinolone acetonide. These results are in favor of using herbal treatments.

Salvizan gel is an almost new product in the market of Iran. Before, two studies evaluated the effects of Salvizan gel on oral diseases. In 2021, Ghalayani et al. [24] reported that Salvizan gel performed better than triamcinolone acetonide in decreasing pain intensity of oral lichenoid reactions, but there was no difference in the extent of the lesion between the two drugs. In 2017, Babadi et al. [25] compared the effect of Salvizan Gel with Triadent (triamcinolone acetonide) in patients with minor aphthous ulcers. They recalled and examined patients on days 2 and 6 to evaluate the pain and ulcer size. They concluded that both drugs significantly reduced the amount of pain and the size of the oral aphthous; Salvizan gel had a better performance in controlling pain, and Triadent ointment had a better performance in reducing the size of the ulcer. The present study also showed a significantly better performance of Salvizan gel in pain control. Although it did not evaluate the reduction of the ulcers’ size, it evaluated complete recovery, which also was in favor of Salvizan gel. The differences between the results, might be due to the different methodology (i.e., recall days) or the populations of the studies. Babadi’s study was conducted in Ahvaz, south of Iran. Due to the ethnic differences inside the population of Iran, treatment results might differ among populations of different regions. This study was conducted in Isfahan, in the center of Iran. So, it is also a limitation to generalize the results of this study as it might be different in other populations. Another limitation of the study was the lack of control over drug usage. Since the drug should be used multiple times daily, it is impossible to recall the patient each time. In this study, patients’ use of the drug was checked in each recall session. They were also given a card to remember to take the drug. It is hard to recall patients every day, so wound healing progress was examined in 2 days intervals, which can cause an error of 1 day in recording the exact day of complete recovery.

Heretofore no study has been performed to compare the effectiveness of Sage and triamcinolone acetonide in treating aphthous ulcers in the centric population of Iran, so the present study was performed to make this comparison. The results of the present study showed that Salvizan gel is an excellent option for treating RAS. Due to the promising results of the herbal treatments and their popularity among society, researchers are suggested to conduct more studies to evaluate the effect of Sage or other herbal drugs on recurrent aphthous ulcers, in other populations.

## Conclusion

Salvizan gel is an efficient treatment for aphthous ulcers. Patients were satisfied with form, taste, and smell of both triamcinolone acetonide and Salvizan gel, but Salvizan gel performed better in reducing pain recovery and wound healing time. With these promising results, Salvizan can be considered a potential drug for treating aphthous ulcers.

## Data Availability

The data gathered and analyzed in the current study are kept private due to the preference of the authors but will be available from the corresponding author on reasonable requests.

## List of abbreviations

RAS: Recurrent Aphthous Stomatitis
